# Syncope: An Atypical Presentation of Pulmonary Embolism Secondary to Occult Uterine Malignancy

**DOI:** 10.1155/2018/9141529

**Published:** 2018-07-12

**Authors:** Zulfiqar Qutrio Baloch, Muhammad Ayyaz, Muhammad Hussain, Shabber Agha Abbas, FNU Samreen

**Affiliations:** ^1^Department of Internal Medicine, Brandon Regional Hospital, Brandon, FL, USA; ^2^R-Endocrinology, Hamilton, NJ, USA; ^3^National Institute of Cardiovascular Diseases, Karachi, Pakistan

## Abstract

All syncopal patients who present to the emergency department should be considered for pulmonary embolism (PE) as part of their differential diagnosis. PE presenting as a syncopal episode and associated with occult uterine malignancy is uncommon. Review of the literature indicates that up to 10% of patients with unprovoked venous thromboembolism (VTE) are diagnosed with cancer in the year following that first episode of VTE. In patients suspected of having a PE who do not manifest any source of an embolism require eventual workup to screen for an occult malignancy. Here, we report a 74-year-old female who presented to the emergency department following an unexplained sudden loss of consciousness and eventually was found to have a massive saddle embolus caused by a uterine malignancy-induced VTE.

## 1. Introduction

Syncope is defined as a sudden and transient loss of consciousness due to cerebral hypoxemia and hypoperfusion. It is one of the most frequent causes of emergency department visits [1]. Vasovagal syncope is the most common type of syncope accounting for approximately one-third of all cases [2]. Pulmonary embolism (PE), a leading cause of morbidity and mortality [3], can rarely present in this manner. Although the most common reported symptoms of PE are pleuritic chest pain, tachypnea, and dyspnea, syncope represents an atypical presentation of PE [4, 5]. There are various cases of PE-induced syncope reported in the literature which strongly suggests that PE should be kept in the differential when patients present with syncope in the emergency department [6]. Malignancies can initially present with venous thromboembolism (VTE) of major veins including the pulmonary veins [7]. We report a case of a 74-year-old female who presented with syncope due to a PE, which upon further investigation was found to have been caused by an underlying occult uterine malignancy.

## 2. Case Report

A 74-year-old female with no past medical history presented to the emergency department following sudden loss of consciousness while sitting on the couch witnessed by her husband. The patient was unresponsive for one minute and regained consciousness spontaneously. Postsyncopal symptoms included weakness and dyspnea, but she denied any associated chest pain or palpitations. Her husband also denied witnessing any tonic-clonic movement and urinary or fecal incontinence during the episode. Upon arrival of the paramedics, the patient was alert and oriented. Her vitals at the time of evaluation were blood pressure 80/62 mmHg, heart rhythm regular, tachycardia with a rate of 123 beats/minutes, respiratory rate of 23 breaths/minute, and a room oxygen saturation of 89%. She was given an intravenous fluid bolus and transferred to the emergency department. Upon arrival, her blood pressure improved to 94/71 mmHg, but she remained tachycardic at a rate of 120 beats/minutes with a respiratory rate of 21 breaths/minute and an oxygen saturation of 92% on 2 L nasal cannula. Physical examination of her head and neck was normal. Chest wall examination was normal without any abnormal movement or tenderness. Patient's lungs were clear to auscultation bilaterally, and no wheezing or crackles were appreciated. Heart and abdominal examinations were unremarkable. Examination of extremities was normal without any edema or signs of a deep venous thrombosis (DVT).

Arterial blood gas investigation revealed hypoxemia (pH: 7.40, pCO_2_: 28, and pO_2_: 61). Her levels of serum electrolytes, glucose, blood urea and creatinine, and complete blood counts were normal. Computed tomographic (CT) scan of her head was negative for any bleeding, embolism, or aneurysm. Her chest X-ray was clear. An electrocardiogram showed a regular rhythm with sinus tachycardia and diffuse T-wave inversion in leads II, III, AVF, and V1 to V6.

CT angiography of the lung demonstrated a large saddle embolus bridging the main pulmonary arteries with extensive segmental and subsegmental clot burden bilaterally, greatest within the left lower lobe but seen within all segments of both lungs. Evidence of right heart strain with flattening and mild leftward bowing of the interventricular septum was also discovered. A transthoracic echocardiogram showed normal left ventricle function without patent foramen ovale, atrial septal defect, or ventricular septal defect. A Doppler scan of the legs showed a DVT involving the distal right femoral vein and right popliteal vein.

Thrombolysis was initiated via catheter-directed therapy. The patient was stable during and after the procedure. Follow-up pulmonary arteriography confirmed resolution of the saddle emboli. The patient was then started on standard anticoagulation treatment with unfractionated heparin and an oral anticoagulant. A blood sample was obtained to study the thrombophilia panel before treatment. An inferior vena cava (IVC) filter was placed without any complication.

Repeated lower extremity ultrasounds confirmed resolution of the DVT. Due to her old age and presence of unprovoked DVT in the leg, she was highly suspicious for an occult malignancy. Abdominopelvic CT scan demonstrated an abnormal appearance of the uterus. There was a rim enhancing mass seen centrally within the uterus with central low attenuation, and additional low attenuation was observed within the endometrial canal. Pelvic ultrasound showed a 3.5 cm uterine mass with increased vascularity. Biopsy of the mass showed early stage endometrial adenocarcinoma. Protein C and S testing of the patient was normal, and the only risk factor for VTE was the uterine malignancy. The patient was discharged five days later on dabigatran, and she reported no further complications or adverse effects. The patient was followed by gynecologic oncology and had laparoscopic removal of the uterine mass. Patient's repeated imaging following the procedures did not show any repeat growth.

## 3. Discussion

VTE often occur following prolonged immobilization such as after major surgeries such as orthopedic surgery [6]. Other potential causes of VTE include familial predisposition or acquired hypercoagulable states such as factor V Leiden deficiency or an underlying occult malignancy (e.g., pancreatic, ovarian, and uterine cancer) [8, 9]. Patients diagnosed with idiopathic VTE (commonly referred to as unprovoked VTEs) are often found to have underlying occult malignancy responsible for the thromboembolism as was the case with our patient [4].

PE itself represents a common cause of death worldwide [3]. PE is difficult to diagnose in many cases due to varied signs and symptoms as the frequently reported classic triad, that is, dyspnea, chest pain, and hemoptysis may not occur in every case [5]. According to the literature, patients with PE can either have submassive or massive embolism. In submassive PE, patients can have symptoms due to pulmonary infarction such as pleuritic chest pain and hemoptysis, or they can present with unexplained dyspnea. With massive PE, patients often present with signs and symptoms of acute cor pulmonale, that is, syncope, cardiac arrest, or sudden death [5, 10]. When patients with PE present with atypical symptoms such as cough, hypotension, and syncope as in our case, diagnosis can be delayed which severely affects the management and prognosis of the patient. Our patient experienced a massive embolism obstructing the main pulmonary artery which resulted in a severe drop in blood pressure which was likely responsible for the syncopal episode.

Syncope can be the initial presentation in up to 10% of patients with PE although it may not be recognized as such at the time of presentation [11]. The consequences of a massive PE presenting in such an atypical manner are catastrophic. Prandoni et al. conducted a cross-sectional study in which they performed a systematic workup for PE in a large series of patients who were hospitalized for a first episode of syncope [12]. They found a high prevalence of PE among their patients, involving approximately one of every six patients (17.3%) in their study. Interestingly, although 25% of patients with PE had presented with syncope of undetermined origin as is expected, almost 13% of patients with PE were considered to have potential alternative explanations for their syncope. Other studies that attempted to report the prevalence of this patient population seemingly underestimated the true prevalence since diagnostic testing was performed only in selected subgroups [13, 14].

Given that syncope is not frequently associated with a PE, physicians may tend to ignore pursuing workup relative to such an etiology [15]. Many mechanisms have been proposed as responsible for syncope in patients with PE; however, three mechanisms have been more frequently discussed in the literature: acute right heart failure, arrhythmia due to right heart overload, and vasovagal reflex [3, 11]. Other cases reported in the literature have suggested that PE should always be in the differential for every syncopal episode encountered in order to decrease associated morbidity and mortality [8, 11, 15].

The association of VTEs with malignancies is well appreciated, and any unprovoked VTE is recognized as a potential initial presentation of an occult malignancy. According to the literature, up to 10% of patients with unprovoked VTE are diagnosed with cancer within the year following their first unprovoked VTE. Despite this recognition, screening strategies having favorable sensitivity and specificity are not available for detecting underlying occult malignancies. A recent randomized, controlled trial conducted by Carrier et al. failed to demonstrate any clinically significant benefit of using abdominal and pelvic CT scans for screening patients for occult malignancies following their first unprovoked VTE [9]. More comprehensive research including both clinical and preclinical research may in the future elaborate upon the existence of such screening methodologies.

## 4. Conclusion

This case was written to report an atypical presentation of PE-induced syncope due to an occult malignancy in an unsuspecting elderly female patient. The clinical picture of PE is variable, which accounts for the frequent failure to identify its presentation, especially in asymptomatic patients or those with atypical presentations. Physicians should retain life-threatening PE amongst their differential diagnosis considerations especially in patients suspected of having an occult malignancy such as elderly patients. The need for rapid diagnosis and treatment in such settings is paramount because with appropriate treatment the majority of patients may survive. Since unprovoked VTA represents a frequent initial presentation of cancer in many patients, it is prudent to screen any such patient for PE. In our case, a patient presenting with syncope, without a significant risk factor for VTE, was found to have underlying uterine malignancy responsible for the development of a massive PE.
